# Editorial: Nutrition and metabolism in musculoskeletal disorders

**DOI:** 10.3389/fnut.2023.1269939

**Published:** 2023-08-23

**Authors:** Mao Zhang, Bo Shan, Sien Lin, Jiankun Xu, Ning Zhang

**Affiliations:** ^1^Stanford Cardiovascular Institute, Stanford University, Stanford, CA, United States; ^2^Institute of Translational Medicine, Zhejiang University, Hangzhou, China; ^3^Department of Orthopaedics and Traumatology, The Chinese University of Hong Kong, Hong Kong, Hong Kong SAR, China; ^4^The Sir Yue-Kong Pao Cancer Centre, Prince of Wales Hospital, The Chinese University of Hong Kong, Hong Kong, Hong Kong SAR, China

**Keywords:** nutrition, metabolism, osteoporosis, sarcopenia, rotator cuff injury, musculoskeletal diseases

Musculoskeletal disorders due to aging, trauma, injury, malnutrition, or other causes are posing significant global challenges, causing mortality and morbidity, in particular to the elderly population (1). The role of metabolic and nutritional factors in the onset and progression of these conditions, including dietary changes, physical inactivity, metabolic syndrome, diabetes, hypertriglyceridemia, and obesity (2), has been increasingly recognized. Balanced metabolism shows great importance for the homeostasis of the musculoskeletal system. For instance, hyperglycemia can worsen osteoarthritis and increase fracture risk in diabetes. Diet and nutrients, which can exert pro- or anti-inflammatory effects, also influence musculoskeletal health. Despite their impact, the importance of maintaining a healthy diet and nutritional status for musculoskeletal health is often underestimated, and the role of metabolism in musculoskeletal disorders remains elusive. To address these gaps, researchers have explored the impact of nutrients, such as lipids, vitamins, and minerals, on the musculoskeletal system, aiming to develop innovative therapeutic approaches to tackle musculoskeletal disorders more efficiently.

The Research Topic includes six research studies, providing comprehensive insights into the complexities of nutrition and metabolism in musculoskeletal disorders and unveiling novel therapeutic strategies. These studies have greatly enhanced our understanding of how nutrients and metabolic factors can prevent, manage, and even reverse musculoskeletal diseases.

Nutrients, such as vitamins, minerals, proteins, and fatty acids, are essential for maintaining the homeostasis of bone, muscle, and joint. Adequate dietary consumption of vitamin D, calcium, magnesium, and protein is important for bone strength and preventing osteoporosis. Furthermore, the impact of pro- and anti-inflammatory metabolites from the diet has been studied in the prevention and management of rheumatoid arthritis (3). An anti-inflammatory Mediterranean diet supplemented with omega-3 fatty acids is recommended as an adjustment to the medical treatment (4). Studies have also explored dietary interventions, such as the potential of prunes (dried plums) to modulate inflammatory pathways and mitigate bone loss in postmenopausal women suffering from osteopenia and osteoporosis (5). Additionally, emerging research have highlighted the influence of the gut microbiome on musculoskeletal health (6, 7), with dysbiosis serving as a biomarker of bone metabolic activity (7).

Three papers under the present Research Topic explored the relationship between dietary factors and musculoskeletal disorders. Vitamin B family (B6, B12, folate) is crucial for the metabolism of homocysteine (Hcy), a key risk factor for cardiovascular diseases and obesity. Observational studies show inconsistent links between vitamin B, Hcy, body composition, and musculoskeletal disorders. To establish causal relationships, Fu et al. selected independent single nucleotide polymorphisms of Hcy, vitamin B12, vitamin B6, and folate from Genome-Wide Association Study (GWAS) data to conduct a Mendelian randomization study. They found that higher Hcy levels were robustly associated with musculoskeletal disorders, while higher vitamin B12 level was dramatically associated with decreased body fat percentage, suggesting the causal effects of vitamins and Hcy on fat and musculoskeletal disorders, respectively. Grili et al. investigated the impact of dietary selenium intake on bone mineral density (BMD) in postmenopausal women and observed the highest dietary consumption of Se among women with normal BMD. Women with higher selenium consumption were also less likely to develop osteoporosis. These findings shed light on the potential protective role of selenium in preventing osteoporosis and provide valuable insights for dietary interventions to improve bone health in postmenopausal women. The role of dietary consumption of S-Equol, a metabolite of soy or daidzein produced by gut bacteria, was explored by Xu et al. in diabetic osteoporosis (DOP). The results showed that with a 12-week intervention, S-Equol improved bone metabolism and enhanced BMD in a streptozotocin-induced DOP model. S-Equol also protected osteoblasts from high glucose treatment in cellular models. Using pharmacological inhibitors and ERβ siRNAs, the authors showed that S-Equol tightly regulated the ratio of OPG/RANKL, a key player in the osteoclast differentiation (8), via the ERβ-PI3K/AKT axis. The above findings suggested that dietary supplements of vitamin B, selenium, and S-Equol have the potential to be adjuvant for treating musculoskeletal disorders.

Obesity, characterized by excess lipid levels and adipose tissue (AT), leads to dyslipidemia and AT remodeling, which can impact musculoskeletal health. In this Research Topic, three papers cover the above aspects. Ge et al. examined the effects of HIF1α inactivation-induced healthy AT remodeling in a model of sarcopenic obesity. Inactivation of adipocyte HIF1α improved AT metabolic health, reduced serum lipid levels and pro-inflammatory cytokines, and a significant reduction in muscle inflammation in obese mice fed with a high-fat diet. Additionally, the administration of the adiponectin receptor agonist AdipoRon mimicked the protective effects against muscle inflammation. These findings suggest that promoting healthy AT remodeling could be a potential therapeutic approach to enhance muscle health in sarcopenic obesity.

The rest two papers under this Research Topic investigated the correlation between dyslipidemia and musculoskeletal disorders. Huang Z. et al. conducted a case retrospective study involving 302 Chinese patients to assess the impact of serum lipid levels on lumbar spine degeneration. They found that age, high-density lipoprotein, and triglycerides affected the degree of degeneration in patients with symptomatic lumbar degeneration without underlying diseases. Age and body mass index were two major factors affecting the severity of degeneration in patients with underlying diseases, with dyslipidemia serving as a secondary factor. Leveraging lipidomic methodology, Huang H. et al. quantitatively analyzed lipid species to identify five lipid molecules [17:1 Lyso PI, 18:0–22:6 PE, 18:3 (*cis*) PC, 22:0 Lyso PC, and 24:0 Lyso PC] closely related to rotator cuff tear and two lipid metabolites (24:0 SM and 16:0 ceramide) related to fatty infiltration severity. However, as these two studies were based on a Chinese population with small sample sizes, their conclusions should be confirmed in other populations or large studies in the future.

In conclusion, this Research Topic has significantly advanced our understanding of the interplay between nutrition, metabolism, and musculoskeletal health. By elucidating the links between nutrients and musculoskeletal disorders, these studies have offered innovative insights into preventive measures and treatment strategies. Tailored nutritional interventions and metabolic modulation have the potential to revolutionize orthopedic surgery, rehabilitation, and management of musculoskeletal disorders. We encourage the scientific community to build upon these foundations and foster collaborative efforts to drive further advancements in the field. With ongoing research and multidisciplinary collaborations, we are confident to pave the way toward optimized musculoskeletal health for all individuals.

## Author contributions

MZ: Writing—original draft, Writing—review and editing. BS: Writing—review and editing. SL: Writing—review and editing. JX: Writing—review and editing. NZ: Writing—original draft, Writing—review and editing.

## References

[1] Briggs AM Cross MJ Hoy DG Sanchez-Riera L Blyth FM Woolf AD. Musculoskeletal health conditions represent a global threat to healthy aging: a Report for the 2015 World Health Organization World Report on Ageing and Health. Gerontologist. (2016) 56:S243–55. 10.1093/geront/gnw002 26994264

[2] Berenbaum F Wallace IJ Lieberman DE Felson DT. Modern-day environmental factors in the pathogenesis of osteoarthritis. Nat Rev Rheumatol. (2018) 14:674–81. 10.1038/s41584-018-0073-x 30209413

[3] Coras R Murillo-Saich JD Guma M. Circulating pro- and anti-inflammatory metabolites and its potential role in rheumatoid arthritis pathogenesis. Cells. (2020) 9:827. 10.3390/cells9040827 32235564 PMC7226773

[4] Nikiphorou E Philippou E. Nutrition and its role in prevention and management of rheumatoid arthritis. Autoimmun Rev. (2023) 22:103333. 10.1016/j.autrev.2023.103333 37182439

[5] Damani JJ De Souza MJ VanEvery HL Strock NC Rogers CJ. The role of prunes in modulating inflammatory pathways to improve bone health in postmenopausal women. Adv Nutr. (2022) 13:1476–92. 10.1093/advances/nmab162 34978320 PMC9526830

[6] Rodrigues FG Ormanji MS Heilberg IP Bakker JL de Borst MH. Interplay between gut microbiota, bone health and vascular calcification in chronic kidney disease. Eur J Clin Invest. (2021) 51:e13588. 10.1111/eci.13588 33948936 PMC8459296

[7] Seely KD Kotelko CA Douglas H Bealer B Brooks AE. The human gut microbiota: a key mediator of osteoporosis and osteogenesis. Int J Mol Sci. (2021) 22:9452. 10.3390/ijms22179452 34502371 PMC8431678

[8] Soos B Szentpetery A Raterman HG Lems WF Bhattoa HP Szekanecz Z. Effects of targeted therapies on bone in rheumatic and musculoskeletal diseases. Nat Rev Rheumatol. (2022) 18:249–57. 10.1038/s41584-022-00764-w 35273387

